# Examining the Utility of the Mammalian Methylation Array for Pan-Mammalian Analysis of Monozygotic Twinning

**DOI:** 10.3390/epigenomes8040037

**Published:** 2024-10-06

**Authors:** Jenny van Dongen, Charles E. Breeze, Twinning Genetics Consortium

**Affiliations:** 1 Department of Biological Psychology, Vrije Universiteit Amsterdam, Van der Boechorststraat 7, 1081 BT Amsterdam, The Netherlands; 2 Amsterdam Reproduction and Development Institute, 1081 HV Amsterdam, The Netherlands; 3 UCL Cancer Institute, University College London, 72 Huntley Street, London WC1E 6BT, UK

**Keywords:** identical twins, epigenetics, genetics, clonal species, comparative epigenomics

## Abstract

Background/Objectives: Human identical twins are born at a rate of 3–4 per 1000 live births. Many other mammals also occasionally produce monozygotic twins, referred to as sporadic polyembryony. The underlying mechanisms are unknown. Through epigenome-wide association studies (EWAS), we identified a robust DNA methylation signature in somatic tissues from human monozygotic (MZ) twins, comprising 834 differentially methylated positions (MZ-DMPs). The results point to a connection between monozygotic twinning and early genome programming and enable new angles to study monozygotic twinning. Methods: The mammalian methylation array (MMA) measures 38,608 CpGs focusing on regions that are well-conserved across many mammalian species, allowing for pan-mammalian comparative epigenomic studies. Here, we successfully map human MZ-DMPs to probes of the mammalian methylation array across 157 mammalian genomes. Results: As expected, based on the modest probe overlap between Illumina 450k/EPIC and mammalian methylation array probes, only a subset of MZ-DMPs reside in conserved regions covered by the mammalian methylation array. These include probes mapping to *NPAS3*, *KLHL35*, *CASZ1*, and *ATP2B2*. Re-analysis restricting the original EWAS in humans to conserved MMA regions yielded additional MZ-DMPs, suggesting that more loci may be detected by application of the mammalian array to monozygotic twins. Conclusions: In conclusion, the mammalian methylation array may prove to be a promising platform to study whether a shared DNA methylation signature of sporadic polyembryony exists across diverse mammalian species. This may potentially point to shared underlying mechanisms.

## 1. Introduction

The production of multiple embryos from a single fertilized egg cell, also called polyembryony, is a relatively common reproductive strategy in plants and invertebrate animals, but it is rare in vertebrates. Humans are monotocous mammals that usually give birth to one child. Twins are born occasionally, with identical (monozygotic) twins occurring in 3–4 per 1000 live births [1,2]. Monozygotic twins are believed to arise from splitting of the embryo in the first two weeks after conception, but the underlying mechanisms remain largely unknown. Human monozygotic twin pregnancies are associated with increased risk of malformations, spontaneous abortion, still birth, and pregnancy complications [3]. A widely accepted, but unproven, hypothesis states that the time of splitting determines the chorionicity of monozygotic twins [4]: splitting at the 2- to 8-cell stage (day 1–3) is believed to give rise to dichorionic diamniotic monozygotic twins (1/3 of all human monozygotic twins); the most commonly found monochorionic diamniotic monozygotic twins (2/3) arise from splitting of the inner cell mass between day 3 and 8; and monochorionic monoamniotic monozygotic twins (<1%) separate at the late blastocyst stage. Even later splitting is believed to result in conjoined twins. The process of splitting has occasionally been recorded through (time-lapse) imaging of human IVF embryos and illustrates that multiple distinct process can underly the formation of human monozygotic twins [5]. These include blastomere separation as early as the 2-cell stage [6], inner cell mass splitting [7], and atypical (“eight-shaped”) hatching [8].

Although often described as ‘a human trait’, monozygotic twinning has been relatively well documented in farm animals such as pigs [9], sheep [10], and cattle [11] after large-scale genotyping was introduced. Case reports exist for many other species [12,13,14,15]. This indicates that sporadic (accidental) polyembryony is taxonomically widespread in vertebrates and affects both monotocous species that typically produce one offspring per pregnancy (including humans) and polytocous species where larger litters are the norm. Unique to placental mammals, facultative polyembryony is exhibited by certain species of armadillo of the *Dasypus* genus, including the nine-banded armadillo (*Dasypus Novemcinctus*). Nine-banded armadillo always produce monozygotic quadruplets [16]. At present, the polyembryonic process in armadillos, which involves post-implantation splitting after a prolonged period of delayed implantation, to our knowledge, has only been described in this species [17]. The mechanisms behind sporadic monozygotic twinning in humans and other species remain to be uncovered [5].

We recently identified a robust DNA methylation signature in somatic tissues from human monozygotic twins, comprising 834 differentially methylated positions (MZ-DMPs), enriched for loci with roles in embryonic processes, cell adhesion, and metastable epi-alleles whose epigenetic state is thought to be established early in development and subsequently mitotically inherited [18]. These findings enable new angles to study monozygotic twinning. At present, no studies to date have examined DNA methylation in monozygotic multiples from species other than humans. Recently, the mammalian methylation array was developed to allow for comparative analysis of DNA methylation across mammals. The array measures up to 38,608 CpGs focusing on highly conserved DNA sequences across many mammalian species [19,20]. These CpGs were selected because they reside in highly conserved DNA sequences and are well conserved across many mammalian species. Here, we successfully map differentially methylated positions (MZ-DMPs) identified in human monozygotic twins to the mammalian methylation array across 157 mammalian genomes and discuss potential opportunities for pan-mammalian DNA methylation analyses of monozygotic (MZ) twinning.

## 2. Results

**Figure 1:**
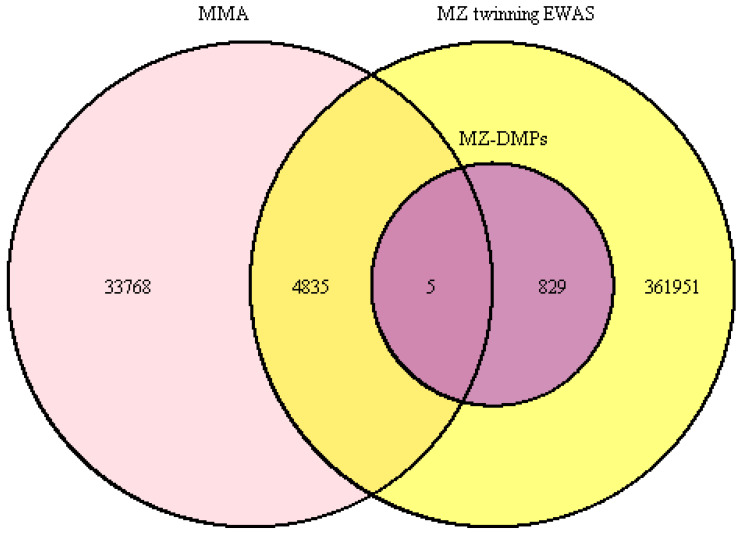
Overlap between methylation sites interrogated by the mammalian methylation array and methylation sites tested in the EWAS of human monozygotic twinning. MZ twinning EWAS = methylation sites tested in the EWAS meta-analysis of human monozygotic twinning (autosomal methylation sites that were present after QC in all twin cohorts). MMA = methylation sites interrogated by the mammalian methylation array. MZ-DMPs = differentially methylated positions in human monozygotic twins.

Figure 1 illustrates the overlap between the 367,620 methylation sites from the EWAS meta-analysis of human MZ twining based on Illumina 450k/EPIC arrays and 38,608 methylation sites interrogated by the mammalian methylation array. In total, 4840 methylation sites are shared between the human EWAS and the mammalian methylation array, representing 1.3% of human EWAS sites and 12.5% of mammalian methylation array sites. Of the 834 epigenome-wide significant MZ-DMPs, five methylation sites are interrogated by the mammalian methylation array (representing 0.6% of all MZ-DMPs). These CpGs map to genes including *NPAS3*, *KLHL35*, *CASZ1*, and *ATP2B2* (Table 1). Although the percentage of MZ-DMPs covered by the mammalian methylation array (0.6%) is smaller than the background percentage of human Illumina 450k/EPIC array probes that overlap with the mammalian methylation arrays (1.3%), MZ-DMPs were not significantly depleted on the mammalian methylation array (X^2^ = 3.308, df = 1, *p*-value = 0.06895).

For the five MZ-DMPs interrogated by the mammalian methylation array, species-specific mapping information was analyzed. Out of all studied mammals, five species (3.2%) showed successful mapping of all 5 CpGs; all of them were primates, and 91 species from 14 taxonomic orders (representing 58% of all examined species, and covering 70% of examined taxonomic orders) showed four mapped CpGs. Mammalian methylation array probes were carefully selected based on alignment across close to 100 different vertebrate genomes. Nevertheless, successful mapping does not always imply that the probes map to the same gene as in humans. Table 2 shows the number of species exhibiting 0, 1, 2, 3, 4, or 5 human MZ-DMP probes mapping to human orthologous genes. In 24 species (32% of species with information on orthologous gene mapping) from six taxonomic orders (43%), at least four CpGs mapped to human orthologous genes (Table 3). These species belong to the following orders: Artiodactyla (four species, namely, sheep, cattle, domestic pigs, and domestic goats), Carnivora (four species, including domestic cats and domestic dogs, Canadese lynx, and American mink), Cetacea (one species, namely, Bottlenose dolphins), Perissodactyla (one species, namely, donkeys), Primates (11 species), and Rodentia (three species, namely, common degus, Damaraland mole rats, and thirteen-lined ground squirrels).

Annotation data for nine-banded armadillos are shown in Table 4 and illustrate successful probe sequence matching to the nine-banded armadillo genome for four CpGs. Two CpGs map to human orthologous genes (cg15089111, *NPAS3*, and cg10816626, *CASZ1*), the first of which also maps to the same region in both species (exon). This table also includes additional potential model organisms, which have been reported to produce monozygotic multiples, namely, cattle [11], pigs [21], mice [22], dogs [13], and sheep [10,23]. In cattle, pigs, and dogs, four out of the five MZ-DMPs map to human orthologous genes. In mice, and sheep, three out of the five MZ-DMPs map to human orthologous genes.

Restricting the human EWAS meta-analysis of MZ twinning to the overlapping CpG sites from the mammalian methylation array and the human llumina 450k/EPIC array (equivalent to an EWAS on a subset of mammalian conserved regions) yielded a total of nine methylation sites significant after multiple testing correction for 38,608 tests. Human annotation data for these CpGs are provided in Table 5. Note that this analysis does not actually cover all 38,608 conserved CpGs interrogated by the mammalian methylation array, but only the 4840 overlapping CpGs measured by both arrays.

## 3. Discussion

The mammalian methylation array was developed to enable high-throughput comparative analysis of DNA methylation in conserved genomic regions across mammals. We explored the overlap of human MZ-DMPs identified through EWAS based on human Illumina 450k/EPIC arrays with mammalian methylation array probe content, and the mapping of these probes to 157 mammalian genomes. As expected, based on the modest overlap of Illumina 450k/EPIC probes and mammalian methylation array probes, only a small proportion of MZ-DMPs resides in mammalian conserved regions covered by the mammalian methylation array. These include probes mapping to *NPAS3*, *KLHL35*, *CASZ1*, and *ATP2B2*. Re-analysis of the original EWAS meta-analysis of human MZ twinning restricting to mammalian methylation array probes yielded additional DMPs at the mammalian array significance threshold, suggesting that additional differentially methylated loci may reside within mammalian conserved genomic regions. Additional DMPs were mapped to different loci including *HOXA5*, *KSR2*, and *HCCA2*/*DUSP8.* Some of these loci have obvious roles in embryonic development, including *HOXA5* (homeobox transcription factor with a key role in morphogenesis [24]) and *CASZ1* (a zinc finger transcription factor with crucial roles in tissue differentiation [25]). *ATP2B2*, a member of the plasma membrane calcium ATPase family that encodes a calcium pump, has been described as a blastocyst upregulated gene in pig embryos [26].

Comparative genomic analysis of 157 mammalian genomes revealed that the number of MZ-DMP probes that map to human orthologous genes is highest (100%) in several primate species. Furthermore, a large number of taxonomically diverse species show orthologous gene mapping for the majority of MZ-DMP probes, including both monotocous and polytocous species. Two of the five MZ-DMPs map to human orthologous genes in the nine-banded armadillo. A limitation of this analysis is that the comparison is based only on a small number of MZ-DMPs, which means that results should be interpreted with caution. Although only a handful of currently identified human MZ-DMPs are interrogated by probes on the mammalian methylation array, additional differentially methylated loci may be detected through pan-mammalian comparison of DNA methylation between monozygotic/polyembryonic individuals versus singleton individuals from different species. At present, such data are not yet available. The mammalian methylation consortium has released DNA methylation data from 15,456 samples from 348 mammalian species. The large majority of individuals can be assumed not to be monozygotic multiples and, thus, serve as controls for such a future analysis. Such analysis is warranted to shed light on the shared mechanisms underlying sporadic polyembryony across vertebrates and shared molecular signatures of sporadic polyembryony and facultative polyembryony. Knowledge on the processes underlying monozygotic twin formation is limited. Inner cell mass duplication has, to our knowledge, only been recorded in humans [27,28]. Atypical blastocyst hatching (“eight-shaped hatching”) has been recorded in humans [8,29], mice [30], and cattle [31]. The presumed unique process underlying the formation of monozygotic quadruplets in nine-banded armadillos has been described in this species only [17]. In nine-banded armadillos, after implantation of the blastocyst, the ICM develops into one amnion and one epiblastic plate and a cavity forms between the implantation site and the amnion. The epiblastic plate differentiates into separated embryonic shields, each capable of developing into an individual. Subsequently, the cavity expands, and it is the physical force of expansion that splits the four embryonic shields and drives it to separate locations, producing identical quadruplets.

At present, it is not yet known when the DNA methylation signature of monozygotic twins is established. Enrichment of MZ-DMPs in binding sites for early-expressed transcription factors, involved in embryonic pattern formation [18], and presence of the signature in blood and buccal cells (derived from different embryonic cell layers), suggests that DNA methylation for at least a subset of loci is established in the early embryo. Future studies in additional human tissues, including pre- and perinatal tissues can provide more information on when and in which cell lineages MZ-DMPs are established. The DNA methylation signature of human monozygotic twinning has at present not yet been studied in placenta, but this would be highly valuable because of (1) the importance of this tissue to the risks associated with monozygotic twin pregnancies due to unequal placental sharing or vascularization and (2) the insight it can provide as to whether DNA methylation differences are present in the trophectoderm-derived extraembryonic cell lineage. In addition, cross-species comparisons may provide more insight. DNA methylation changes driven by an atypical prenatal environment of twins are less likely to be shared with other animals that present different in utero developmental conditions. For example, in polytocous animals, which typically give birth to >= 2 offspring, competition for space and nutrients is less of an issue. Furthermore, different factors, such as genetic background, in-utero exposures, and maternal diet are very different across species. Thus, detecting a conserved DNA methylation signature of monozygotic twinning across species would eliminate unknown human-specific confounders as an alternative explanation, and add to the evidence that DNA methylation is connected to the process of embryonic splitting.

Application of the mammalian methylation array to diverse mammalian species has previously proven successful for deriving pan-mammalian epigenetic clocks, which are DNA methylation-based estimators of chronological age that exploit the very strong relationship between an organism’s age and DNA methylation. While this principle was originally detected in humans, the same principle was later proven to also apply to taxonomically diverse non-human mammalian species [32], and even to amphibia [33] and fish [34]. In fact, a single equation can predict age across all mammals using DNA methylation data [32,35]. Although the exact CpGs that perform best in age prediction can sometimes be species-specific, these findings demonstrate the existence of an evolutionary conserved pattern of DNA methylation tracking chronological age. In a similar fashion, investigating if monozygotic twins from other non-human species also exhibit a somatic DNA methylation signature may point to a shared underlying mechanism responsible for the DNA methylation signature of monozygotic twins.

One limitation of this work is genomic coverage by DNA methylation arrays used in humans (Illumina450k/EPIC), which only measure a small proportion of CpGs in the genome. The small number of MZ-DMPs interrogated by the mammalian methylation array, as well as the small number of (nine-banded) armadillos with mammalian methylation array data available at the moment, currently precludes cross-species comparisons of human monozygotic twins to nine-banded armadillos. The comparative genomic analysis was restricted to mammals with NCBI reference genomes available. Future reference genome data from additional species would allow for even more comprehensive comparisons. Although monozygotic twinning has been described in taxonomically diverse species, for the large majority of species in the animal kingdom, it is still an open question whether monozygotic twins occur. This question could be answered through genotyping of twins or larger litters. For monotocous animals, the lack of knowledge on twinning is related to the rareness of the event and likely high mortality rate of twins, making observations of twins difficult (especially in wild animals); if twins are observed, they would then also need to be genotyped to establish zygosity. For polytocous animals, monozygotic twins among similar-looking littermates will go unnoticed, and, again, genotyping is the only reliable method to establish zygosity. Even for frequently genotyped farm animals in breeding programs, such as pigs, it is not common practice to genotype entire litters, while this could yield valuable information on the frequency of monozygotic twinning.

Although the frequency of sporadic monozygotic twinning is largely unknown for most species, frequencies have been recorded and are of comparable orders of magnitude to humans for some species, including chimpanzee [36] and cattle [11], and appear to be higher in pigs [9,21,37]. Although sporadic monozygotic twins are born at low rates, samples from monozygotic multiples may be collected in sufficient numbers from species that are bred in high numbers, such as farm animals and laboratory animals such as mice. Importantly, the rate of monozygotic twinning is perceived to be much higher than the number of monozygotic twin births, due to abortion, still birth, and vanishing twins. In this light, it is also relevant that prenatal (or aborted) samples can be more easily obtained in farm animals and laboratory animals, as well as in pets undergoing C-section or selective abortion (as is regular practice in horse breeding when twins are encountered). Finally, conjoined twins, presumed to be (nearly) always monozygotic are readily recognizable, and have been described for many species. In addition to sporadic monozygotic twins, vertebrate species that exhibit facultative polyembryony [38], including the nine-banded armadillo [17], provide access to large numbers of monozygotic individuals to study molecular signatures of polyembryony.

In conclusion, a small proportion of human MZ-DMPs identified through Illumina 450k/EPIC array-based EWAS is interrogated by the mammalian methylation array. The mammalian methylation array may prove to be a valuable platform to study the extent to which DNA methylation signatures of polyembryony are shared across monozygotic multiples from diverse mammal species, which may point to shared underlying mechanisms.

## 4. Materials and Methods

### 4.1. Mammalian Methylation Array

The mammalian methylation array (llumina HorvathMammalianMethylChip40 BeadChip) was developed to perform comparative DNA methylation analysis across mammals [19]. The mammalian methylation array measures up to 38,608 CpGs per species. Although the target (probe) sequence might be conserved, the targeted sequence can map to different genes in different specifies. Mammalian array data generated by the mammalian methylation consortium are available for ~15,000 samples from 348 mammalian species [20]. The mammalian methylation array annotation data for 159 mammals (125 unique species) were downloaded from https://github.com/shorvath/MammalianMethylationConsortium/ (accessed on 25 July 2024). For the remaining 189 mammalian species, no NCBI reference genome data are available yet; hence, they do not have genomic annotation data. Genomic annotation data for 159 mammals included two human genomes (two different genome builds), which were excluded from the current comparison.

### 4.2. Mammalian Species Information

Species information for the 159 mammals described by Arneson et al. [19] including taxonomic order and species common and Latin names were obtained from supplementary dataset 2 from Arneson et al. [19] and from HorvathMammalChip_SpeciesSourcesTaxonomyAndKeys_v1.3.csv provided by the Mammalian Methylation Consortium.

### 4.3. MZ Twinning EWAS Meta-Analysis

We previously performed an EWAS meta-analysis of MZ twinning using the Illumina 450k array and the Illumina EPIC array in MZ and DZ twins [18]. This study used blood samples from 5 independent cohorts (with replication in buccal cells in one cohort). These cohorts were: Netherlands Twin Register (NTR), Environmental Risk Longitudinal Twin Study (E-Risk), Finnish Twin Cohort (FTC), UK Adult Twin Registry (TwinsUK), and Brisbane Systems Genetic Study (BSGS). The EWAS results were presented in human genome build hg19. In the current analysis, we utilize the genome-wide summary statistics from this EWAS: 367,620 CpGs, including 834 CpGs that were differentially methylated in MZ twins at epigenome-wide significance level after Bonferroni correction for the Illumina 450k/EPIC array common probes. We refer to these as MZ-DMPs (differentially methylated positions in monozygotic twins).

### 4.4. Analysis

To examine how many of the 834 previously identified human MZ-DMPs are interrogated by the mammalian methylation array, the overlap between MZ-DMPs and the mammalian methylation array was assessed based on CpG identifier, which indicates probe fidelity between arrays. To test if MZ-DMPs are over- or underrepresented on the mammalian array CpGs, a chi-squared test was performed (R function chisq.test). As background, we utilized the 367,620 CpGs meta-analyzed in our EWAS of MZ twinning [18]. Second, for each of the 157 non-human mammalian species, we assessed whether MZ-DMP probes interrogated by the mammalian methylation array mapped to the target species genome. Third, we compared the aligned regions in each target species with human, based on the Ensemble orthologue gene identifiers for human and the target species, as described in Arneson et al. [19]. This information was taken from the column “conservationInHuman”, available for 76 species. No mammalian methylation array data have been generated yet on human monozygotic twins, but to explore whether additional MZ-DMPs might be detected in mammalian conserved DNA, we repeated the EWAS meta-analysis of MZ twinning (based on Illumina 450k/EPIC arrays), restricting to the probes that overlap with the mammalian array, applying a Bonferroni-corrected alpha for conserved region EWAS; 0.05/38,608 = 1.3^−6^. Note that this analysis does not actually provide full coverage of mammalian conserved regions due to the limited overlap of the arrays. eFORGE-40k (https://eforge40k.altiusinstitute.org/, accessed on 16 May 2024) was used to analyze human DHS data of MZ-DMPs [39,40,41].

## Figures and Tables

**Table 1:** Characteristics of MZ-MPs that are interrogated by the mammalian methylation array.

IlmnID	N	Z-Score	*p*-Value	CHR	Position	Human Gene	Human Nearest Gene
cg15089111	5722	−5.884	3.995 × 10^−9^	14	34270113	*NPAS3*	*NPAS3*
cg16547529	5723	5.594	2.223 × 10^−8^	11	75140681	*KLHL35*	*KLHL35*
cg10816626	5722	−5.514	3.509 × 10^−8^	1	10711457	*CASZ1*	*CASZ1*
cg14209399	5720	−5.409	6.349 × 10^−8^	3	10370507	*ATP2B2*	*ATP2B2*
cg02170386	5723	−5.397	6.759 × 10^−8^	14	70316972		*SMOC1*

The table shows CpGs that were identified in a large EWAS of MZ twinning utilizing Illumina EPIC/450k array data after Bonferroni correction, covered by the mammalian methylation array. N = total meta-analysis sample size (number of twins). Z-score = meta-analysis effect size (positive values correspond to higher methylation level in monozygotic twins and negative values correspond to lower methylation level in monozygotic twins). CHR = Chromosome. Human genomic annotation information is given in genome build 37. Note: cg02170386 is located in an intergenic region in the human genome. Because this CpG does not map directly to a gene in humans, the column “human gene symbol” is empty.

**Table 2:** Number of MZ-DMPs mapped to human orthologous genes across taxonomic orders.

Order	N Species	N MZ-DMP Probes Mapped to Human Orthologous Genes						
		0	1	2	3	4	5	Median
Cetacea	1	0	0	0	0	1	0	4
Perissodactyla	1	0	0	0	0	1	0	4
Primates	19	0	0	3	5	9	2	4
Artiodactyla	8	0	0	0	4	4	0	3.5
Carnivora	12	1	1	3	3	4	0	3
Chiroptera	3	0	0	1	2	0	0	3
Cingulata	1	0	0	1	0	0	0	2
Lagomorpha	1	0	0	1	0	0	0	2
Monotremata	1	0	0	1	0	0	0	2
Proboscidea	1	0	0	1	0	0	0	2
Rodentia	24	0	4	9	8	3	0	2
Dasyuromorphia	1	1	0	0	0	0	0	0
Didelphimorphia	1	1	0	0	0	0	0	0
Diprotodontia	2	2	0	0	0	0	0	0
N total	76	5	5	20	22	22	2	

The table shows, for each order, the number of species that show 0, 1, 2, 3, 4, or 5 MZ-DMP probes that map to a human orthologous genes, where 5 is the maximum. The second column shows the total number of species per order with genomic annotation data available for the mammalian methylation array. Only species with information on orthologous gene mapping are included in this table. The final column shows the median number of MZ-DMP probes mapped to human orthologous genes across all species belonging to a certain order. The table is sorted by median; orders with the highest order median human orthologous gene mapping are on top.

**Table 3:** Mammalian species with ≥80% of mammalian array-covered MZ-DMP probes mapped to human orthologous genes.

Species Latin Name	Common Name	Order	N Mapped ^a^	N Human Orthologous Gene ^b^	N Human Orthologous Gene and Region ^c^
Rhinopithecus bieti	Black-and-white snub-nosed monkey	Primates	5	5	2
Saimiri boliviensis	Black-capped squirrel monkey	Primates	5	5	2
Gorilla gorilla	Gorilla	Primates	5	4	1
Canis lupus familiaris	Dog	Carnivora	4	4	1
Capra hircus	Domestic goat	Artiodactyla	4	4	1
Felis catus	Cat/Domestic cat	Carnivora	4	4	2
Bos taurus	Cattle	Artiodactyla	4	4	1
Cebus capucinus	Colombian white-faced capuchin	Primates	4	4	2
Colobus angolensis	Angola colobus	Primates	4	4	2
Equus asinus	Donkey	Perissodactyla	4	4	1
Cryptomys damarensis	Damaraland mole rat	Rodentia	4	4	1
Ictidomys tridecemlineatus	Thirteen-lined ground squirrel	Rodentia	4	4	1
Lynx canadensis	Canada lynx	Carnivora	4	4	1
Mandrillus leucophaeus	Drill	Primates	4	4	1
Microcebus murinus	Grey mouse lemur	Primates	4	4	1
Neovison vison	American mink	Carnivora	4	4	1
Octodon degus	Common degu	Rodentia	4	4	1
Papio anubis	Olive baboon	Primates	4	4	1
Pan paniscus	Bonobo	Primates	4	4	1
Sus scrofa	Domestic pig	Artiodactyla	4	4	2
Rhinopithecus roxellana	Golden snub-nosed monkey	Primates	4	4	2
Ovis aries	Sheep	Artiodactyla	4	4	2
Theropithecus gelada	Gelada	Primates	4	4	1
Tursiops truncatus	Bottlenose dolphin	Cetacea	4	4	1

The table shows all species for which at least 4 out of the 5 MZ-DMP probes map to a human orthologous gene. ^a^ Number of MZ-DMPs mapped to the species’ genome. ^b^ Number of MZ-DMPs mapped to human orthologous gene. ^c^ Number of MZ-DMPs mapped to human orthologous gene and to the same gene region as in humans.

**Table 4:** Genomic annotation of mammalian array-interrogated MZ-MPs for putative model organisms.

Species	CpG	cg15089111	cg16547529	cg10816626	cg14209399	cg02170386
*Homo Sapiens*	Chr	14	11	1	3	14
	Position	34270113	75140681	10711457	10370507	70316972
	Human gene symbol	*NPAS3*	*KLHL35*	*CASZ1*	*ATP2B2*	
	Human nearest gene	*NPAS3*	*KLHL35*	*CASZ1*	*ATP2B2*	*SMOC1*
	DHS Tissue	ES cell	Blood, Fetal Heart, Fetal Kidney, Fetal Muscle Leg, Fetal Stomach	ES cell, Fetal Adrenal Gland, Fetal Brain, Fetal Lung	ES cell, IPS cell	Fetal Brain, Fetal Muscle Leg, Placenta, Psoas Muscle
*Dasypus novemcinctus*	CpG	cg15089111	cg16547529	cg10816626	cg14209399	cg02170386
	Conservation class	1. conserved gene and region	NA	2. conserved gene but different region	3. mapped to different genes	3. mapped to different genes
	Human gene symbol	*NPAS3*	*KLHL35*	*CASZ1*	*ATP2B2*	
	Dasypus novemcinctus gene symbol	NPAS3	NA	CASZ1	GHRL	SLC10A1
*Bos taurus*	CpG	cg15089111	cg16547529	cg10816626	cg14209399	cg02170386
	Conservation class	1. conserved gene and region	NA	2. conserved gene but different region	2. conserved gene but different region	2. conserved gene but different region
	Human gene symbol	*NPAS3*	*KLHL35*	*CASZ1*	*ATP2B2*	
	Bos taurus gene symbol	NPAS3	NA	CASZ1	ATP2B2	SMOC1
*Mus musculus*	CpG	cg15089111	cg16547529	cg10816626	cg14209399	cg02170386
	Conservation class	NA	NA	2. conserved gene but different region *	1. conserved gene and region	2. conserved gene but different region
	Human gene symbol	*NPAS3*	*KLHL35*	*CASZ1*	*ATP2B2*	
	Mus musculus gene symbol	NA	NA	Casz1	Atp2b2	Smoc1
*Sus scrofa*	CpG	cg15089111	cg16547529	cg10816626	cg14209399	cg02170386
	Conservation class	1. conserved gene and region	NA	2. conserved gene but different region	1. conserved gene and region	2. conserved gene but different region
	Human gene symbol	*NPAS3*	*KLHL35*	*CASZ1*	*ATP2B2*	
	Sus scrofa gene symbol	NPAS3	NA	CASZ1	ATP2B2	SMOC1
*Canis lupus familiaris*	CpG	cg15089111	cg16547529	cg10816626	cg14209399	cg02170386
	Conservation class	1. conserved gene and region *	NA	2. conserved gene but different region	2. conserved gene but different region	2. conserved gene but different region
	Human gene symbol	*NPAS3*	*KLHL35*	*CASZ1*	*ATP2B2*	
	Canis lupus familiaris gene symbol	NPAS3	NA	CASZ1	ATP2B2	SMOC1
*Ovis aries*	CpG	cg15089111	cg16547529	cg10816626	cg14209399	cg02170386
	Conservation class	1. conserved gene and region	NA	2. conserved gene but different region	2. conserved gene but different region	3. mapped to different genes
	Human gene symbol	*NPAS3*	*KLHL35*	*CASZ1*	*ATP2B2*	
	Ovis aries gene symbol	NPAS3	NA	CASZ1	ATP2B2	SLC10A1

* These entries were manually corrected as the original annotation file misclassified the conservation class based on ensemble orthologous IDs. DHS = probe mapping to tissue-specific Dnase 1 hypersensitive sites. ES cell = embryonic stem cell. IPS cell = induced pluripotent stem cell. NA indicates that the probe CpG does not map to the target species genome.

**Table 5:** Characteristics of the top differentially methylated sites in human monozygotic twins that are interrogated by the mammalian methylation array.

IlmnID	N	Z-Score	*p*-Value	CHR	Position	Human Gene	Human Nearest Gene
cg15089111 *	5722	−5.884	3.995 × 10^−9^	14	34270113	*NPAS3*	*NPAS3*
cg16547529 *	5723	5.594	2.223 × 10^−8^	11	75140681	*KLHL35*	*KLHL35*
cg10816626 *	5722	−5.514	3.509 × 10^−8^	1	10711457	*CASZ1*	*CASZ1*
cg14209399 *	5720	−5.409	6.349 × 10^−8^	3	10370507	*ATP2B2*	*ATP2B2*
cg02170386 *	5723	−5.397	6.759 × 10^−8^	14	70316972		*SMOC1*
cg04863892	5722	5.260	1.439 × 10^−7^	7	27183375	*HOXA5*	*HOXA-AS3*
cg16300531	5722	−5.093	3.529 × 10^−7^	12	118405988	*KSR2*	*KSR2*
cg02005600	5723	4.880	1.059 × 10^−6^	7	27183686	*HOXA5*	*HOXA-AS3*
cg05280206	5722	−4.848	1.246 × 10^−6^	11	1575607	*HCCA2*; *DUSP8*	*MOB2*

* MZ-DMPs; CpGs that were epigenome-wide significant in the MZ twinning EWAS meta-analysis of human Illumina EPIC/450k array data after Bonferroni correction. N = total meta-analysis sample size (number of twins). Z-score = meta-analysis effect size (positive values correspond to higher methylation level in monozygotic twins and negative values correspond to lower methylation level in monozygotic twins). The table shows all CpGs that are interrogated by both the mammalian array and the Illumina 450k and EPIC array and that have a *p*-value < 1.3 × 10^−6^ in the human Illumina EPIC/450k meta-analysis (Bonferroni significance threshold for the mammalian methylation array). Human genomic annotation information is provided (genome build 37).

## Data Availability

The summary statistics from the human EWAS of monozygotic twinning are available at https://www.nature.com/articles/s41467-021-25583-7 (accessed on 20 May 2024) [18]. The mammalian array annotation data are available at https://github.com/shorvath/MammalianMethylationConsortium/ (accessed on 20 May 2024); for more information see the original publications [19,20].
